# Binding of Red Clover Isoflavones to Actin as A Potential Mechanism of Anti-Metastatic Activity Restricting the Migration of Cancer Cells

**DOI:** 10.3390/molecules23102471

**Published:** 2018-09-26

**Authors:** Grażyna Budryn, Joanna Grzelczyk, Horacio Pérez-Sánchez

**Affiliations:** 1 Institute of Food Technology and Analysis, Faculty of Biotechnology and Food Sciences, Lodz University of Technology, 90-924 Lodz, Poland; joanna.grzelczyk@edu.p.lodz.pl; 2 Bioinformatics and High-Performance Computing Research Group (BIO-HPC), Computer Engineering Department, Universidad Católica de Murcia (UCAM), Guadalupe, 30107 Murcia, Spain; hperez@ucam.edu

**Keywords:** actin, cell migration, isoflavones, anti-metastatic activity

## Abstract

Actin functions are crucial for the ability of the cell to execute dynamic cytoskeleton reorganization and movement. Nutraceuticals that form complexes with actin and reduce its polymerization can be used in cancer therapy to prevent cell migration and metastasis of tumors. The aim of this study was to evaluate the ability of isoflavones to form complexes with actin. Docking simulation and isothermal titration calorimetry were used for this purpose. The formation of complexes by hydrogen bonds, hydrophobic and π-π interactions was demonstrated. Interactions occurred at the ATP binding site, which may limit the rotation of the actin molecule observed during polymerization and also at the site responsible for contacts during polymerization, reducing the ability of the molecule to form filaments. The greatest therapeutic potential was demonstrated by isoflavones occurring in red clover sprouts, i.e., biochanin A and formononetin, being methoxy derivatives of genistein and daidzein.

## 1. Introduction

Actin is one of the most abundantly occurring proteins in the human body. In muscle cells, it makes up 10% and in other cells from 1% to 5%. Actin appears as a globular monomer (G-actin) but undergoes rapid reorganization and polymerizes forming filaments (F-actin), which makes actin the basic biomolecule that controls most cell processes and functions; including the shape, adhesion, and motility [1]. Aside from the polymerization of actin, mechanisms involved in these transformations also require complexing with numerous cellular proteins. The phenomenon of cell migration in tissue organisms has a twofold significance. It is necessary for the wound healing process [2,3] but it also contributes to the increase in motility and invasion of cancer cells [4]. Metastasis accounts for over 90% of cancer-related mortality, highlighting the need to increase the understanding of the regulatory mechanisms responsible for cell motility and targeting cancer cell migration is of great importance. The structure of actin allows the attachment of ligands that can limit or completely block cellular functions. The molecular mass of actin is 43 kDa and it consists of 375 amino acids divided into two domains [5]. The smaller domain is further divided into subdomains 1 and 2, and the larger into subdomains 3 and 4. Subdomain 1 contains residues 1–32, 70–144, and 338–372, and subdomain 2 contains residues 33–69, including the DNase I loop with residues 38–52. Subdomain 3 contains residues 145–180 and 270–337 and subdomain 4 contains residues 181–269 [6]. The attachment of P_i_ to ADP results in conformational changes of the DNase I loop and a shift of domain 2. The differences between the conformational states are relatively small and include interactions with SER14 in subdomain 1 [1]. The nucleotide binds to the upper gap between domains 1, 3, and 4 through hydrogen bonds at residues 14–18, 157–159, 213–214 and 302, and hydrophobic interactions at 302, and 305–306. The cellular functions of actin result from the ability of monomers to polymerize into filaments. In G-actin, the two main domains are located parallel to one other. The F-actin, formed as a result of polymerization, has a helical structure. The initial step of polymerization requires rotation between domains 1 and 3. The link between these domains is relatively small, through the polypeptide chain that forms the hinge with residues including Tyr143, Ala144, Gly146, Thr148, Gly168, Ile341, Ile345, Leu346, Leu349, Thr351, Met355 and Cys374, which are predominantly hydrophobic. These form stable linkages with larger molecules of typical toxins [7], which causes a drastic loss in the rotation and polymerization ability, blocking cell functions.

Binding of nutraceuticals to actin at other sites is one potential mechanism that can limit actin polymerization, thus preventing cancer cell movement and cancer invasion. So far, little progress has been made in the search for compounds that disrupt the organization of actin filaments, although it is already known that cytoskeletal reorganization caused by the binding of ligands to actin allows not only the inhibition of migration but also apoptosis of cancer cells [8,9,10]. By binding to actin, nutraceuticals can modify and determine the intracellular location of transcription factors that play important roles in gene transcription [11,12]. The binding ability of actin active sites has been demonstrated for many nutraceuticals, including flavonoids. One special family of flavonoids are isoflavones, the source of which are leguminous plants. Genistein and daidzein are the two most-studied isoflavones, due to their abundant presence in soybeans [13,14]. Another very important source of isoflavones is red clover, which, in addition to soy, is used for the production of phytoestrogen preparations. Clover contains high concentrations of two other isoflavones, formononetin and biochanin A, and their content increases rapidly when sprouts are harvested. Clover isoflavones have been shown to have a strong affinity for estrogen receptors, including ERβ [15]. Potentially, they can also bind to actin, limiting its ability to polymerize and form complexes with other proteins. The aim of this study was to evaluate the affinity of isoflavones to bind to actin by docking simulation (DS) and isothermal titration calorimetry (ITC). Isoflavones other than genistein were studied for binding with actin for the first time and ITC was used for the first time to study the interactions of actin with potential drugs.

## 2. Results and Discussion

### 2.1. Concentration of Isoflavones in Red Clover Sprouts

Red clover sprouts were cultivated until the 11th day, after which they had become seedlings. White, UVA and UVB light was used for cultivation. Every day, samples were taken for the analysis of isoflavones and then from each lighting condition those sprouts were selected, which were characterized by the highest content of isoflavones. In the case of UVA, two local maxima of total isoflavone concentration were observed on the 3rd and 8th day of cultivation, which was not observed under UVB or white light, where the concentrations gradually increased to the 10th (white light) or 11th (UVB) day of cultivation. Therefore, two trials grown under UVA were selected for the ITC tests. The sprouts were dominated by methoxy derivatives, especially formononetin (from 496.15 mg/100 g of dry basis (d.b.) in spouts cultivated for 11 days under UVB to 1449.87 mg/100 g d.b. in sprouts cultivated for 10 days under white light), followed by biochanin A (from 152.10 to 353.15 mg/100 g d.b. in sprouts cultivated for 8 days under UVA and for 11 days under UVB) (Table 1). 

Lower levels of daidzein and genistein were found in the sprouts. The highest concentrations of these isoflavones were recovered from sprouts cultivated under UVB. In terms of the isoflavone content, the most preferred was the use of white light, which resulted in a content of these compounds at almost 2% d.b., which makes them a good candidate for the preparation of dietary supplements for mitigating the effects of menopause [15].

### 2.2. Characterisation of Complexes of Isoflavones with Actin

In our studies, we determined the activity of isoflavones against metastases of tumors. The prophylactic or therapeutic effects of dietary components can be tested using different models. The simplest is related to interactions between a biomolecule, most commonly a protein or nucleic acid, and a ligand, which is a component of the diet. These are simple models that can be expanded using more complex systems, for example on cell lines or in animal studies.

The biomolecule-ligand models include thermodynamic studies using the isothermal titration calorimetry (ITC) method. This method allows for the determination of a binding constant, enthalpy and entropy of interaction as well as free enthalpy, i.e., the affinity of a ligand to a biomolecule. Few available studies have compared the results of interactions obtained using the ITC method with another model of the biomolecule-ligand interaction, namely molecular modelling by docking simulation (DS). This method allows determination of affinity as well as the site and type of interactions [16]. Each model provides different and complementary information about the occurrence and nature of interactions. 

Our study concerned interactions between actin and isoflavones as potential ligands changing the ability of the protein to polymerize from the G to F form. Limiting the polymerization by binding active sites or by changing the conformation may reduce cancer cell migration and metastasis [8]. Polyphenols, including isoflavones, are potential actin ligands, although only genistein has been studied to date [11]. The two most abundant sources of isoflavones in the diet are soy and red clover. The former contains genistein and daidzein, mainly in the form of glycosides, while clover contains biochanin A and formononetin, these being methoxy derivatives of genistein and daidzein. While glycosides must be hydrolyzed by the microbiota to aglycones to be absorbed by the gastrointestinal tract, the methoxy forms are absorbed directly and relatively quickly [17].

ITC analysis showed that a high actin binding constant was determined for biochanin A and formononetin, 2.87 × 10^3^ and 2.41 × 10^3^ L/mol respectively, while genistein and daidzein showed a lower binding constant of 0.82 × 10^3^ and 1.34 × 10^3^ L/mol respectively (Table 2). Xiao and Kai [18] showed that the methylation of the hydroxyl group of flavonoids increases their affinity to serum proteins by several times. Our studies showed a similar relationship with the cytoskeleton protein. Thus, actin complexes with red clover isoflavones are more stable and their biological effect may potentially be more long-lasting compared to soy isoflavones. In other studies using the tryptophan quenching fluorescence method, Böhl [11] determined the binding constant of genistein to actin at 3.50 × 10^4^ L/mol and quercetin in these studies had a higher *K_A_* of 4.61 × 10^4^ L/mol; similarly to taxifolin 4.41 × 10^4^ L/mol. Although the *K_A_* determined by quenching tryptophan differs from our results, it shows that the potential of isoflavones to bind with actin is similar to that of known drugs. With the binding of quercetin to actin Böhl, Czupalla, Tokalov, Hoflack, and Gutzeit [19] explained the inhibition of bone resorption by this substance.

**Figure 1:**
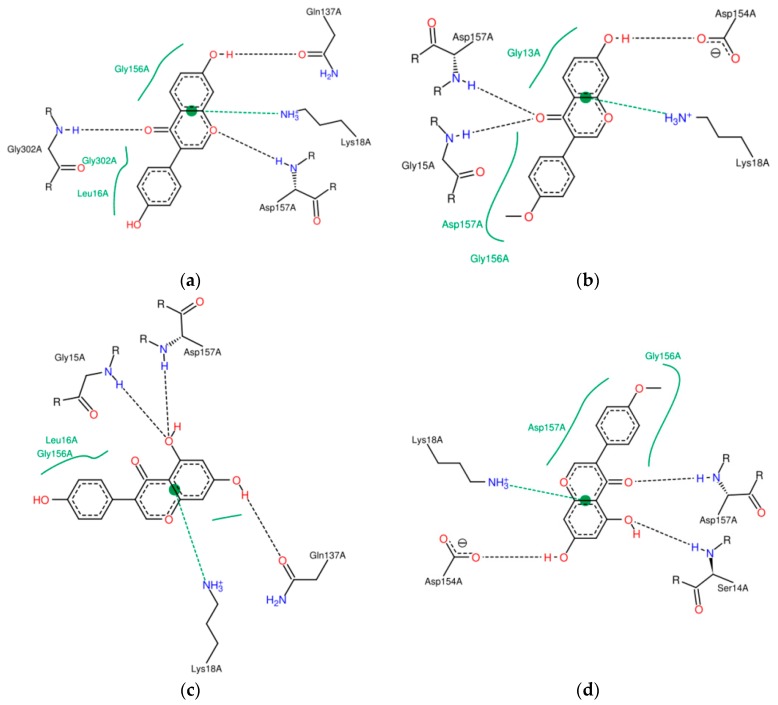
Depiction (in 2D) of the main interactions established between the active site of actin and (**a**) genistein; (**b**) formononetin; (**c**) daidzein; (**d**) biochanin A. Continuous lines represent hydrophobic interactions, while dashed lines show hydrogen bonds and dashed lines connected to the bond between ring A and C represent π-π interactions.

Differences in *K_A_* may result from the number and position of binding sites and from the type of interactions. The ATP binding region is involved in interactions that enable fitting and formation of the polymer. Binding of an isoflavone to the ATP binding site of actin could inhibit the polymerization of the protein because ATP binding could be competitively inhibited by the isoflavone [11]. All tested isoflavones formed complexes at the nucleotide binding site, including Ser14, Gly15, Leu16, Lys18, Asp157, Glu214, Gly302, MET305, Tyr306 (Figure 1). 

The isoflavones analyzed did not interact with the hinge helix between subdomains 1 and 3 (Figure 1). Blocking this hinge could cause a drastically toxic effect. The rotation between subdomains 1 and 3 means that the side chain of Glu137 can combine with ATP, enabling its hydrolysis during polymerization [20]. Genistein and daidzein interacted through hydrogen bonds with Glu137, which may moderately decrease the ability of actin to form filaments (Figure 1a,c) [6,21].

The methylation of the C4′ hydroxyl group in the biochanin A molecule in relation to genistein caused a shift of the hydrogen bond with Asp157 from the hydroxyl group at C5 to the carbonyl at C4 and hence the additional stabilization through hydrogen bonding of the C ring (Figure 1c,d). Hydrophobic interactions in both complexes were related to other amino acids but involved the B-ring of these isoflavones. In turn, formononetin in relation to daidzein also has a methoxy group at C4′, which resulted in the presence of two hydrogen bonds at the oxygen of the carbonyl group at C4 and the absence at O1 observed only for daidzein. Furthermore, formononetin in relation to biochanin A, and daidzein in relation to genistein, does not have a hydroxyl group at C5, so that in formononetin and daidzein an additional hydrophobic interaction at the A ring was possible (Figure 1a,b). According to Böhl [11], hydrophobic interactions are more important for reducing the functionality of actin. Nevertheless, the greater stability of complexes in the case of two methoxy derivatives, in particular when they interact together with actin in one system, may result from forming complementary hydrogen bonds with the Gly13, Ser14 and Gly15 residues in subdomain 1.

Daidzein and genistein differ in the presence of the hydroxyl group at C5 in the latter molecule. At the hydroxyl group in the genistein molecule, two hydrogen bonds with Gly15 and Asp157 occurred. Xiao [18] noted that an additional hydroxyl group in the C-ring of flavonoids may attenuate protein affinity, especially when it results from hydrophobic interactions. In our studies, an increase in affinity was observed with the occurrence of an additional hydroxyl group in the A-ring.

This may be due to the fact that the actin residues located in the binding sites are flexible and can adapt to attach to an isoflavone and form additional hydrogen bonds. The absence of the hydroxyl group at C5 in daidzein contributed to the attachment of Asp157 at O1 and to additional hydrophobic interactions at Gly302. According to Dedova [6], these two amino acids are part of the actin fragment also responsible for binding the nucleotide. Böhl [11] ascribed the ability of flavonoids to inhibit actin polymerization by complexing at, or near, the ATP binding site. All the analyzed isoflavones possessed a π-π-type linkage with Lys18, which stabilizes the interactions and this side chain is involved in binding ATP [6].

### 2.3. Energetic Effects of Actin-Isoflavones Interactions

**Figure 2:**
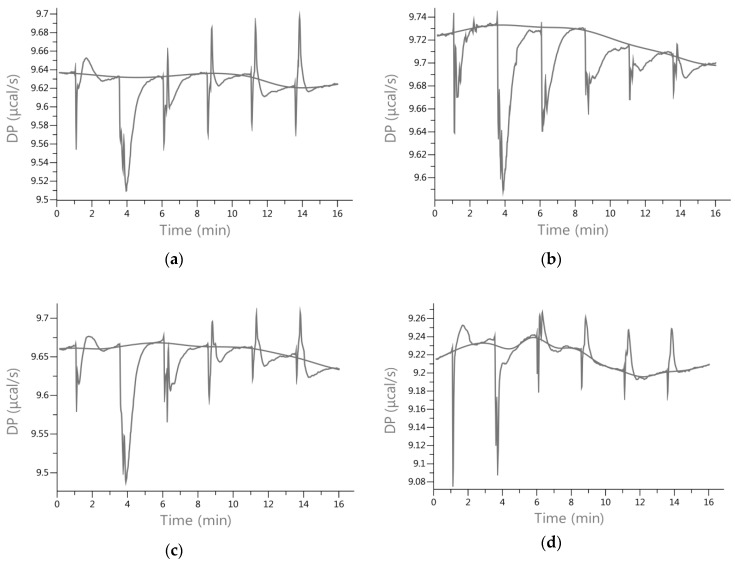
ITC raw data from the titration of actin with red clover sprout extracts cultivated under (**a**) white light for 10 days; (**b**) UVA 3 for days; (**c**) UVA for 8 days; (**d**) UVB for 11 days.

The obtained enthalpies of the complexation indicate a different nature of interactions that yielded both exo- and endothermic effects (Table 2). The interactions of actin with biochanin A and genistein were endothermic (Δ*H* = 23.49 and 23.03 kJ/mol respectively, *p* > 0.05). According to Frazier, Papadopoulou, and Green [22] as well as Du, Li, Xia, Ai, Liang, and Sang [23], this endothermic effect may be due to a conformational rearrangement in the protein molecule by disruptions of the energetically favorable noncovalent interactions. As shown by Dominguez [20], the actin molecule is flexible and changes the conformational structure as a result of molecular interactions. Flat flavonoids, including isoflavones, require stearic adaptation of actin and hence cause conformational changes and disrupt its functions. Thus, the effects of complexing actin with isoflavones are not only interactions at the active sites that result in blocking binding other ligands or enabling polymerization but also conformational changes limiting the transcriptional mechanisms depending on actin allosteric effects. In the case of the titration of actin with isoflavone pairs, the exothermic nature of complexation was observed in the case of biochanin A with daidzein and daidzein with formononetin. Other pairs showed moderate exothermic effects of binding with actin. Clear exothermic effects were shown by actin titration with red clover sprout extracts (Figure 2). They were similar and their amount ranged from −22.59 to −21.10 kJ/mol, which showed the great potential of extracts, constituting of a mixture of isoflavones and other bioactive substances, to form complexes with actin. 

The affinity, i.e., the change of free enthalpy during the actin-isoflavone titration, was determined by ITC (Δ*G*) and compared with the affinity calculated by DS (Δ*G_predicted_*) (Table 1). In both methods, the affinity was negative, which indicates the spontaneous nature of the interactions. The free enthalpy determined by the ITC method was in the range from −19.09 to −16.08 kJ/mol, for biochanin A and genistein respectively. In the case of the DS method, the affinity obtained was in the range from −38.49 kJ/mol for biochanin A to −35.98 kJ/mol for daidzein. It was shown that DS more closely reflects the static effects resulting from the docking of ligands, while the ITC also shows the energetic effects arising from protein structure rearrangement. In the evaluated interactions of the actin with pairs of isoflavones, affinity was in the range from −21.14 kJ/mol for formononetin with biochanin A to −15.74 kJ/mol for daidzein with biochanin A, which indicates an advantage of energy effects associated with complexing and blocking the active sites of actin by the mixture of the two methoxy derivatives. 

The nature of the interactions of the four sprout extracts with actin was different (Figure 2). Except for the extract from sprouts grown under UVA for 3 days, all other extracts showed, after the initial exothermic effect associated with binding of isoflavones, in the further part of the titration a smaller or larger advantage of an endothermic effect caused by conformational changes of actin. Despite having the highest content of formononetin and biochanin A and a high negative change in enthalpy for complexing with actin observed for the extract of red clover sprout cultivated under white light, the highest affinity for the protein was achieved with extracts from sprouts grown under UVA, followed by UVB cultivation (Table 2). Sprout extracts grown in white light showed the most significant endothermic effect, suggesting the most significant conformational changes of actin (Figure 2), which in addition to complexing with the active sites observed as exothermic changes recorded especially over the first two injections, may limit the ability of actin to polymerize, similar to the extracts cultivated under UV conditions. 

## 3. Materials and Methods 

### 3.1. Chemicals and Reagents

Actin from porcine muscle ~90% lyophilized powder containing Tris, ATP and CaCl_2_, daidzein ≥98%, biochanin A ≥95%, genistein ≥98% and formononetin ≥99%, were purchased from Sigma Aldrich (St. Louis, MO, USA). 

### 3.2. Isothermal Titration Calorimetry

Calorimetric measurements were conducted using a MicroCal PEAQ-ITC200 (Malvern, Worcestershire, UK) isothermal titration calorimeter. Analyses were carried out according to Budryn [15], with some modifications. We had to use very dilute solutions and decided to conduct the analysis with fewer injections and an increased titrant volume. The calorimeter cell (0.2 mL) was filled with degassed 0.1 μmol/L actin solution and 12 μL portions of degassed 10 μmol/L aqueous solutions of isoflavones were injected into the cell. Measurements were carried out at 25 °C with continuous stirring (307 rpm). Isoflavone aliquots were injected at intervals depending on the time value of the observed effects. Measurements of the thermodynamic effects of dilution of isoflavone solutions were additionally carried out to subtract those effects from the actin titration measurements. The effects of the dilution of the actin solution were negligible.

The analysis was carried out for individual isoflavones or pairs of them to capture synergistic effects and also for extracts from red clover sprouts obtained by dissolving in water the lyophilized methanol-water extract of sprouts at concentrations of 10 mg/mL. The concentration of isoflavones in the extracts was about 1.0 mmol/L calculated on formononetin. An example of ITC raw data is shown in Figure 2. The heat released upon the interaction between actin and the isoflavone was recorded over time and the raw data were obtained as a plot of heat flow in kcal/s against time. The integration of each peak yielded a value for the heat released during each injection of the isoflavone solution into the cell filled with actin solution. The plot of these values vs. the molar ratio isoflave-actin was then used to determine the thermodynamic parameters of the interactions. Parameters such as the binding constant (*K_A_*), enthalpy change (Δ*H*), and entropy change (Δ*S*) were calculated from the ITC titration by nonlinear least-squares curve fitting carried out in MicroCal PEAQ-ITC200 software. The free energy change (Δ*G)* was calculated from the Gibbs equation [24].

### 3.3. Cultivation of Sprouts 

The tested material was red clover *(Trifolium pratense* L.), Rosette variety, suitable for germination, supplied by FN (Granum, Wodzierady, Poland). Seed germination was carried out in a climatic chamber with a KBWF 720 (Binder, Tuttlingen, Germany) phytotron system. Cultivation of the sprouts was carried out for 11 days at relative humidity 80% and 25 °C using white light, UVA (340 nm) or UVB (310 nm) light, 24 h/day. Sprouts were cultivated in portions of 15 g in containers with a perforated bottom placed on a tray. On the first day, the seeds were soaked by the addition of 30 mL of water and on the subsequent days sprouts were irrigated to maintain high moisture in the culture. Sprouts were harvested every day of the cultivation. The cultivation was carried out in triplicate under the same conditions and three samples were taken from each container for analysis. The collected sprouted seeds were frozen at −40 °C [15]. 

### 3.4. Extraction and LC-ESI-MS Analysis of Isoflavones

Sprouts were freeze-dried (20 h, 0.340 mbar and 4 h, 0.250 mbar) in a DELTA 1–24 LSC freeze dryer (Christ, Osterode am Harz, Germany) and ground in A 11 mill (IKA Staufen, Germany) laboratory mill with a screen mesh of 2 mm. The extraction was carried out in an E-916 pressure Speed Extractor (Buchi, Essen, Germany) [25]. The extraction temperature was 50 °C and the pressure 10 MPa. A sample of 0.5 g of the milled lyophilized sprouts was placed in the extraction cell with a capacity of 60 mL. One cycle of extraction was performed with a mixture of solvents composed of methanol:water:acetic acid (90/9/1 *v*/*v*/*v*). The resulting extracts were filtered through a syringe cellulose filter with a pore diameter of 0.2 μm. Then, an aliquot of the sample was analyzed by an LC-ESI-MS system. 

The LC-ESI-MS analysis of isoflavones was performed in accordance with Gao, Yao, Zhu and Ren [26] with some modifications using a liquid chromatograph equipped with a SPD M20A diode array detector and quadrupole mass spectrometer 2020 with an ion source of the electrospray type (ESI) in positive scan mode (Shimadzu, Kyoto, Japan). A Kinetex C18 column (5 mm, 150 × 2.1 mm, Phenomenex, Torrance, CA, USA) was used. The column was thermostated at 30 °C. The sample was placed in autosampler and 10 μL was injected. A gradient elution was performed at the mobile phase flow rate of 0.4 mL/min. The elution gradient and the conditions of MS identification were according to Budryn [15].

### 3.5. Molecular Modelling

In order to obtain detailed information at the atomic level about the interactions between the different isoflavones and actin, we carried out molecular modelling studies. We performed docking simulations because their results can provide information on how hydrogen bonds and hydrophobic as well as other types of interactions between bioactive compounds and actin are formed [15]. 

A representative X-ray crystal structure for actin was 1WUA taken from the Protein Data Bank (PDB) database (http://www.rcsb.org/ pdb). The structure is complexed with ATP and Ca^2+^. Next, a full-atom model of the protein was prepared from the PDB structure. Bond orders were assigned, hydrogens were added and cap termini were included with the Protein Preparation Wizard module available in Maestro [27]. The protonation states of all side chains were subsequently defined using PROPKA3.1. The chemical structures of all isoflavone molecules were built up manually and partial charges were calculated using Gasteiger scheme to be used during docking simulations [28].

The docking of isoflavones to the prepared actin structure model was performed with Autodock Vina docking software [29] using the default configuration parameters. The size of the grid box for ligand docking was set to extend 120 Å in each direction from the geometric center of each individual docking simulation. The scoring function in Vina considers the Lennard-Jones term (LJ), hydrogen bonds (H-bonds), electrostatic interactions, hydrophobic stabilization, entropic penalty due to the number of rotatable bonds, and the internal energy of the ligand.

### 3.6. Statistical Analysis

LC-MS and ITC analyses were conducted at least three times for each of three independent cultivations carried out under the same conditions. All the data are shown as the mean value ± standard deviation (SD). In order to assess the normal distribution of the groups, the Shapiro-Wilk test was performed. Additionally, Levine’s test was performed to confirm the homogeneity of variances, followed by a one-way analysis of variance (ANOVA) to compare the results and a Tukey’s test to reveal the pairs of groups that differ with statistical significance in term of the means. Significance was defined at *p* ≤ 0.05. 

## 4. Conclusions

Considering the activity of isoflavones as nutraceutical that forms complexes with actin that are helpful in the prevention of cancer metastases, it should be taken into account whether they are administered as isolated individual substances or as a mixture in functional foods. Evaluation of a single isoflavone as an actin polymerization inhibitor allowed the indication of a few which were characterized by strong interactions with the protein. These were biochanin A and formononetin, which exhibited a high binding constant and high affinity determined ITC and docking simulation. On the other hand, a promising example of a food that may be of therapeutic significance in limiting cancer metastasis is red clover in the form of sprouted seeds, which contain high concentrations of biochanin A and formononetin. They are quickly absorbed in the digestive tract without the need for hydrolysis. Soy sprouts are rich in genistein and daidzein showing a lower affinity for actin. These compounds in soy are mainly in the form of glycosides, limiting their bioavailability. Other components of sprout extracts may also contribute to the activity against actin polymerization. In further studies, we intend to verify the ability of isoflavones to inhibit cancer cell migration in cell cultures tests.

## Figures and Tables

**Table 1:** Concentration (mg/100 g d.b.) of isoflavones in red clover sprouts.

Red Clover Sprouts *	Daidzein	Formononetin	Genistein	Biochanin A	Total
White light 10	40.14 ± 2.38	1449.87 ± 50.92	118.49 ± 4.88	212.08 ± 11.92	1820.58 ± 55.92
UVA3	3.72 ± 0.17	721.37 ± 32.36	84.47 ± 6.39	192.84 ± 6.18	1002.40 ± 38.48
UVA8	11.85 ± 0.36	794.36 ± 35.59	70.81 ± 3.18	152.10 ± 5.74	1348.32 ± 49.09
UVB11	115.35 ± 7.39	496.15 ± 19.61	188.96 ± 6.96	353.15 ± 15.95	1153.61 ± 37.71

* light used for germination and cultivation time in days; ±SD; cultivation was carried out in triplicate and for LC-MS analysis, three samples were taken from each cultivation; values in columns were significantly different at *p* ≤ 0.05.

**Table 2:** Thermodynamic parameters of the interactions of isoflavones with actin determined by ITC and docking simulation (∆*G_predicted_*).

Isoflavone/Red Clover Sprouts *	*K_A_* × 10^3^ (L/mol)	∆*H* (kJ/mol)	∆*S* (J/mol·K)	∆*G* (kJ/mol)	∆*G_predicted_* (kJ/mol)
single isoflavones					
Daidzein	1.34 ± 0.09	−0.04 ± 0.01	57.85 ± 3.12	−17.29 ± 0.71	−35.98
Formononetin	2.41 ± 0.11	0.11 ± 0.02	62.99 ± 4.05	−18.67 ± 0.90 ^b^	−38.07
Genistein	0.82 ± 0.06	23.03 ± 1.38 ^a^	131.11 ± 10.92	−16.08 ± 0.55	−37.66
Biochanin A	2.87 ± 0.15	23.49 ± 1.08 ^a^	142.77 ± 9.15	−19.09 ± 0.87 ^b^	−38.49
pairs of isoflavones					
Daidzein + Formononetin	-	−8.21 ± 0.60	36.03 ± 2.55	−18.59 ± 0.92 ^a^	-
Daidzein + Genistein	-	0.28 ± 0.04	66.50 ± 5.96	−18.88 ± 0.74 ^a^	-
Daidzein + Biochanin A	-	−11.26 ± 0.71	15.55 ± 0.83	−15.74 ± 0.39	-
Formononetin + Genistein	-	0.85 ± 0.07	60.94 ± 5.39 ^b^	−16.71 ± 0.59	-
Formononetin + Biochanin A	-	5.11 ± 0.41	91.10 ± 7.50	−21.14 ± 0.85	-
Genistein + Biochanin A	-	0.15 ± 0.01	62.98 ± 4.08 ^b^	−18.00 ± 0.81 ^a^	-
red clover sprouts extracts					
White light 10	-	−22.57 ± 0.92 ^b^	−35.89 ± 1.18	−12.23 ± 0.43	-
UVA3	-	−22.19 ± 0.78 ^b^	−9.01 ± 0.38 ^a^	−19.59 ± 0.62 ^a^	-
UVA8	-	−21.19 ± 0.65 ^c^	−5.23 ± 0.31	−19.68 ± 0.71 ^a^	-
UVB11	-	−21.10 ± 1.05 ^c^	−9.88 ± 0.43 ^a^	−18.88 ± 0.65 ^a^	-

* light used for germination and cultivation time in days; ±SD; cultivation was carried out in triplicate and for LC-MS analysis three samples were taken from each cultivation; *K_A_*—binding constant; Δ*H*—enthalpy change; Δ*S*—entropy change; Δ*G*—free energy change—total affinity; values with different letters and without any superscript in one column of a sub-table differ with statistical significance (*p* ≤ 0.05).
